# Shifting Sugars and Shifting Paradigms

**DOI:** 10.1371/journal.pbio.1002068

**Published:** 2015-02-17

**Authors:** Mark L. Siegal

**Affiliations:** Center for Genomics and Systems Biology, Department of Biology, New York University, New York, New York, United States of America

## Abstract

No organism lives in a constant environment. Based on classical studies in molecular biology, many have viewed microbes as following strict rules for shifting their metabolic activities when prevailing conditions change. For example, students learn that the bacterium *Escherichia coli* makes proteins for digesting lactose only when lactose is available and glucose, a better sugar, is not. However, recent studies, including three *PLOS Biology* papers examining sugar utilization in the budding yeast *Saccharomyces cerevisiae*, show that considerable heterogeneity in response to complex environments exists within and between populations. These results join similar recent results in other organisms that suggest that microbial populations anticipate predictable environmental changes and hedge their bets against unpredictable ones. The classical view therefore represents but one special case in a range of evolutionary adaptations to environmental changes that all organisms face.

## Abstract 2

This Primer explores three recent PLOS Biology papers that increase our understanding of how microbes respond optimally to the changing availability of nutrients in their environment. Read the Research Articles.

In his doctoral thesis, Jacques Monod described the diauxic shift, whereby a microbial population consumes all of a preferred sugar before metabolizing a less-preferred sugar [1]. The diauxic shift links physiology and ecology and therefore must be understood in terms of the evolutionary fit of an organism to its environment. Although Monod had a deep appreciation of evolution, his standing as one of the great biologists of the modern era stems from his pursuit of the molecular mechanisms underlying the diauxic shift [2]. Indeed, the form in which most students of biology now encounter the diauxic shift is in the canonical example of gene regulation: the *lac* operon of the gut bacterium *Escherichia coli* [3]. Fewer biologists read Monod’s principles of microbial growth under nutrient limitation [4] or stop to reflect on the ecological implications of how microbes regulate their metabolisms [5]. Recent advances in probing metabolic and regulatory mechanisms inside cells, and in surveying natural variation of many species, make the time ripe to revisit the connections between gene regulation, physiology, and ecology.

The *lac* operon, which encodes proteins necessary to metabolize lactose, is a prime example of catabolite repression, which underlies the diauxic shift. Catabolism of glucose leads to a low concentration of cyclic adenosine monophosphate (cAMP). When the catabolite activator protein is bound to cAMP, it binds to DNA and stimulates transcription of the *lac* operon. Thus, the operon is transcribed at a high level only when glucose, the preferred sugar, is absent. *E*. *coli* cells presented with a mix of glucose and lactose will induce the *lac* operon after glucose has been depleted. During this diauxic shift, the population of cells experiences a “lag” (delay) in growth. Monod’s microbiology mentor André Lwoff suggested to Monod that such lags might be caused by the requirement to induce the synthesis of enzymes that had not been needed while glucose was present. Monod, after famously admitting ignorance of the “enzyme adaptation” phenomenon to which Lwoff was referring, showed that Lwoff’s hypothesis was correct [6].

The population lag during diauxic shift has now been observed in many different microbes. For example, when populations of the budding yeast, *Saccharomyces cerevisiae*, are presented with a combination of glucose and galactose, glucose is consumed first, then population growth slows, then exponential growth resumes as galactose is consumed (Fig. 1) [7]. The regulatory mechanism of catabolite repression for this eukaryotic microbe differs from that of *E*. *coli*, but the logic appears to be the same: use the preferred carbon source first, then reconfigure metabolism to use the less-preferred carbon source.

**Fig 1:**
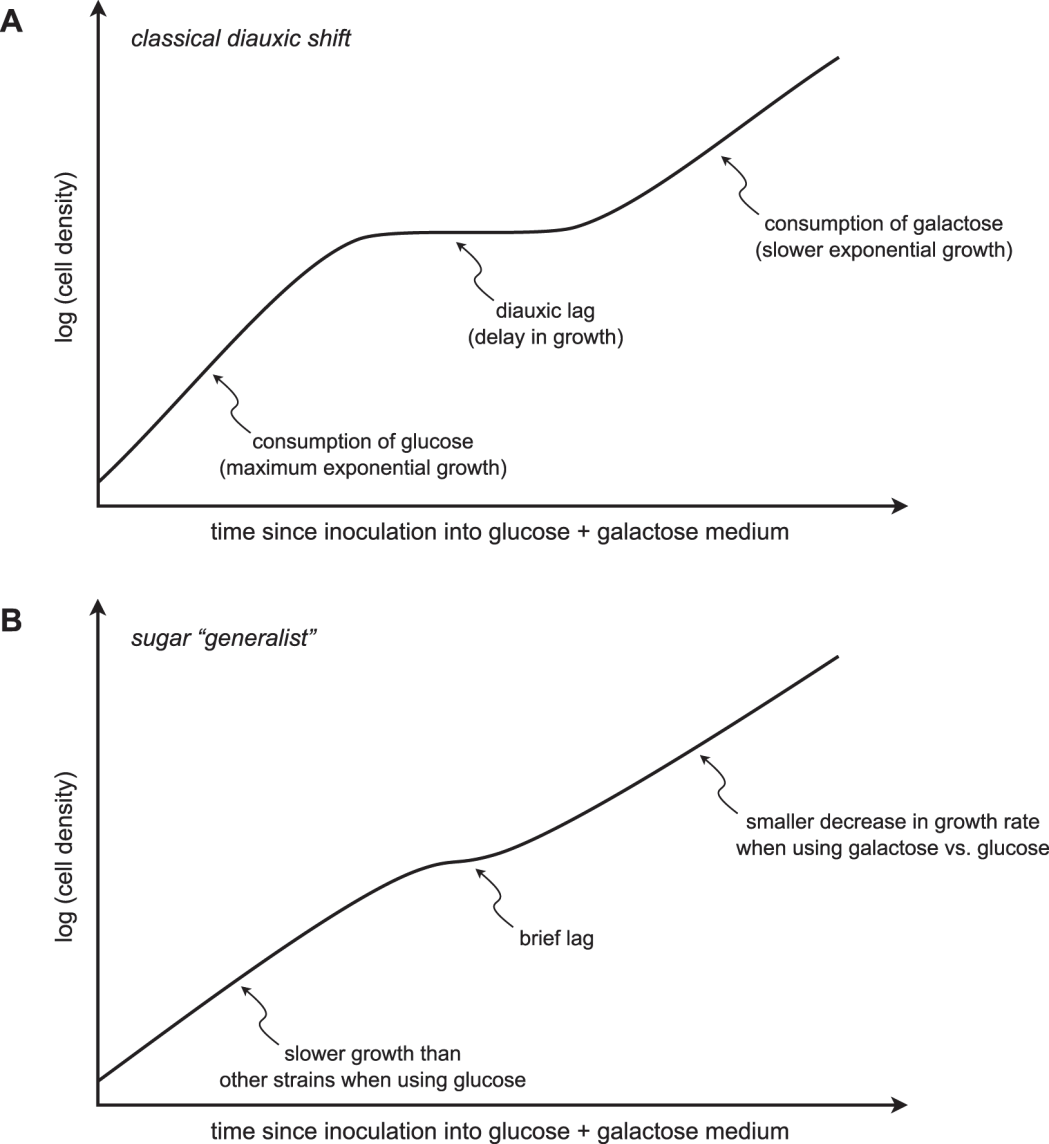
Some strains of budding yeast do not follow the classical diauxic shift. (A) The classical diauxic shift, as introduced by Monod, shows three population-growth phases when cells are presented with a mixture of a preferred sugar and a less-preferred sugar. When a yeast population is presented with a mixture of glucose and galactose, it first grows exponentially at its maximum rate while metabolizing glucose. When glucose is expended, cells cease growth (lag) while activating the galactose utilization pathway. Exponential growth then resumes while the cells metabolize galactose, although growth is slower than when glucose is metabolized. In ecological terms, strains exhibiting this growth behavior can be considered “glucose specialists” [7]. (B) Some strains lag briefly or not at all [7,8]. In such strains, the exponential growth rate while metabolizing galactose tends to be much closer to the exponential growth rate while metabolizing glucose, although the latter growth rate tends to be lower than that of a glucose specialist. Strains exhibiting this growth behavior can be considered sugar “generalists” [7].

Catabolite repression makes intuitive sense as an evolved mechanism for optimizing growth in a changing environment. Indeed, one way students remember the molecular details of the *lac* operon is to consider what the cells “want to do” when, for example, glucose and lactose are both available (despite the cringes this anthropomorphic reasoning should induce, it does work as a memory aide). However, natural environments can be much more complicated than a laboratory environment in which two sugars are presented simultaneously at high concentrations. Three recent papers in *PLOS Biology* tackle the question of how yeast cells respond to different environments in both the physiological and the evolutionary senses [7–9]. They show that there is considerable heterogeneity both within and between populations in the dynamics of sugar utilization and they therefore force a more expansive view of the diauxic shift.

New et al. [7] showed that wild yeast strains differ in their growth responses to different sugar environments in ways that likely reflect different evolutionary pressures. For example, when presented with medium containing a low concentration of glucose and a high concentration of maltose, a less-preferred sugar, some strains obey the classical diauxic shift: they consume glucose, lag for some length of time, then resume growth while metabolizing maltose. However, under identical conditions, other strains do not lag appreciably at all. As New et al. [7] propose, the strains that lag or do not lag can be thought of as ecological “specialists” or “generalists,” respectively. The specialists grow on glucose most efficiently and correspondingly have a high degree of catabolite repression, yet they take a long time to relieve the repression and begin growing when shifted to maltose, on which they do not grow as well. The generalists grow at more similar rates on glucose and maltose, yet their maximum growth rates on glucose are lower than those of specialist strains (Fig. 1) [7]. Ecological theory tells us that generalists should be favored in fluctuating environments, whereas specialists should be favored in relatively stable environments [7]. Experimental evolution bears this out: cycling between glucose and maltose environments tends to select generalist strains with short lags [7].

In a recent issue of *PLOS Biology*, Wang et al. [8] extend the findings of New et al. [7] by confirming important corollaries of Lwoff’s hypothesis. They begin similarly to New et al. [7], by showing that wild yeast strains differ in lag duration when presented with identical mixtures of glucose and galactose [8]. To probe the molecular underpinnings of these differences, they genetically engineered wild yeast strains to express a fluorescent protein under the control of the pathway that induces the genes required to metabolize galactose [8]. A wild strain with a long diauxic lag showed complete catabolite repression, only inducing the fluorescent reporter after exhaustion of glucose [8]. A similar result was seen by New et al. [7] for the maltose pathway in the long-lagging laboratory strain. In contrast, wild strains with shorter diauxic lags induced the fluorescent reporter (and began consuming galactose) before exhaustion of glucose [8]. This result speaks directly to Lwoff’s hypothesis: if a strain does not experience diauxic lag, it should be because the enzymes necessary to metabolize the less-preferred sugar are induced before the preferred sugar is exhausted. In addition, this result suggests that generalist strains should experience a growth rate decrease when grown in conditions under which catabolite repression is incomplete (when glucose levels are low) because they are using energy to express the utilization pathway for the less-preferred sugar even when glucose remains. Wang et al. [8] indeed show that the magnitude of a strain’s growth rate decrease when cultured in a mixture of glucose and galactose (versus glucose alone) positively correlates with the expression of the fluorescent reporter in that mixture.

The finding of Wang et al. [8] that some strains induce the galactose utilization pathway and metabolize galactose before glucose is exhausted implies that these cells have a kind of anticipatory behavior. What form does this anticipation take? Do cells do calculus, measuring the time derivative of glucose concentration, or do they take a current, very low level of glucose as a sign that soon there will be none? Wang et al. [8] find evidence for the latter, in that steady state sugar levels, rather than sugar depletion dynamics, were the key determinant of the extent to which the galactose utilization pathway is induced. Anticipation has been observed in other contexts, such as the evolved “expectation” of *E*. *coli* cells that an increase in temperature (from ambient to human body temperature) will be followed quickly by a decrease in available oxygen [5], or that maltose will be encountered after lactose in the digestive tract [10]. In a fluctuating environment, memory of recent states is a kind of anticipation to the extent that those states are likely to return; such memory, effected by the stable transmission of proteins across generations, is seen in *E*. *coli* cells exposed to an environment fluctuating between carbon sources [11]. Competing negative and positive feedback loops in regulation of yeast galactose utilization can tune memory persistence between a few generations and hundreds of generations [12].

Physiological responses based on robust sensing of prevailing conditions can be contrasted with bet hedging, whereby a population maximizes its long-term fitness in an uncertain environment by generating heterogeneity. Like the diauxic shift, bet hedging is also a longstanding idea in the biological literature that is experiencing a resurgence of interest [13]. The two are not mutually exclusive, and instead are better viewed as lying at opposite poles of a spectrum of strategies evolved to cope with a varying environment [14]. Indeed, both New et al. [7] and Wang et al. [8] observed variability among single cells within strains, in addition to average differences between strains. For example, New et al. [7] found high variability in lag in strains that had high average lag durations. This trend had also been seen in a study that used high-throughput microscopy to track many thousands of individual cells that were returned to a low-glucose environment after having depleted glucose [15]. Likewise, in particular mixtures of glucose and galactose and in particular strains, induction of the galactose utilization pathway is bimodal, with some cells fully inducing and others not inducing at all [8,9,16]. These observations suggest that, although anticipation is possible, there are limits to its accuracy so that some amount of bet hedging is also adaptive [17,18]. As noted by New et al. [7], the adaptive benefit of a combined anticipation and bet-hedging strategy can also be inferred from the finding that their experimentally evolved strains stochastically induce maltose utilization genes in the presence of glucose.

Determining the extents to which sensing and bet-hedging strategies are operating, as well as determining the relevant regulatory mechanisms, requires the ability to probe the physiological responses to many different combinations of environmental variables in individual cells [14,19,20]. Also in a recent issue of *PLOS Biology*, Venturelli et al. [9] advance the state of the art in this respect by using automated flow cytometry to take measurements of fluorescent reporter expression in many cells over fine time courses as yeast populations consume available sugar. Like Wang et al. [8], they use a reporter of the activity of the galactose utilization pathway. They find three distinct behaviors of yeast cell populations, depending on initial glucose and galactose concentrations. When initial glucose levels are sufficiently high, catabolite repression is complete, with no induction of the galactose utilization pathway even at moderately high initial galactose concentrations. When initial glucose levels are sufficiently low and galactose is present, cells uniformly induce the galactose utilization pathway within a short time. However, at many combinations of intermediate initial concentrations of glucose and galactose, populations show bimodal induction of the pathway, with a subset of the population inducing early in anticipation of glucose exhaustion and the rest inducing late [9]. The net result of the bimodality is that the population as a whole shows a reduced lag duration [9]. Moreover, consistent with a bet-hedging view, the advantage of bimodality could be even greater in situations when the environment abruptly changes; by using a microfluidic device to track individual cells and the microcolonies they produce, Venturelli et al. [9] found that cells within a bimodal population that had activated the galactose utilization pathway grew faster upon shift to an environment containing galactose but no glucose, relative to cells that had not activated the galactose utilization pathway. One way to interpret these results is that the cells have the least reliable information about their environment when presented with moderate concentrations of the sugars in combination. Rather than obey the classical diauxic shift and repress the galactose utilization pathway until glucose is expended, some cells make the shift early (perhaps placing a bet on a more acute loss of glucose) (Fig. 2).

**Fig 2:**
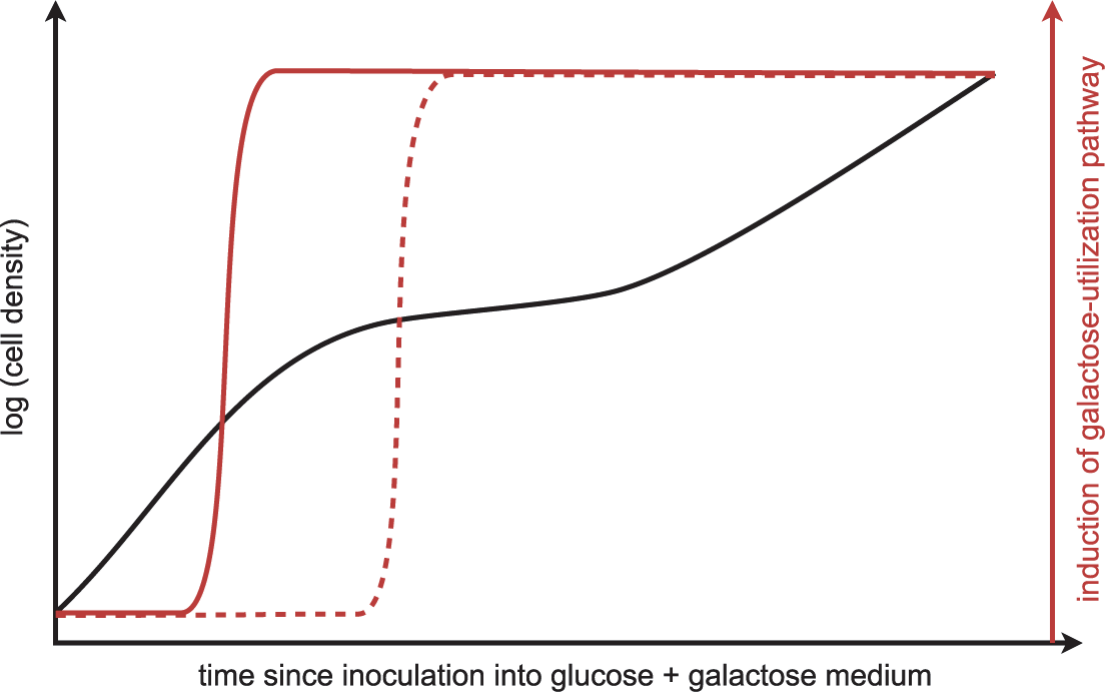
Bimodality in the induction of the galactose utilization pathway can be viewed as a combination of sensing and bet hedging. At the population level, cells inoculated into a mixture of intermediate concentrations of glucose and galactose appear to follow a classical diauxic shift, with two exponential growth phases separated by a lag (black curve). However, this population average behavior might obscure heterogeneity among individual cells. In particular, bimodal responses are seen, in which one subpopulation of cells induces the galactose utilization pathway before glucose is expended (solid red curve), and another subpopulation of cells induces the pathway after glucose is expended (dashed red curve) [9]. Sensing of the sugar environment is clearly involved in this bimodal response, because the late-inducing subpopulation waits for glucose to be expended, and even the early-inducing subpopulation does so only after some time in culture. However, the bimodality suggests the population is hedging its bets. The early inducers can be viewed as bets on abrupt loss of glucose, whereas the late inducers can be viewed as bets on gradual depletion, or even return, of glucose.

Similar bimodal phenomena are seen in other organisms. When presented with a mixture of glucose and cellobiose, populations of the bacterium *Lactococcus lactis* contain one subpopulation that continues to grow after catabolite repression is relieved by glucose exhaustion and another subpopulation that ceases to grow because it does not ever switch to cellobiose metabolism [21]. The latter subpopulation grows faster than the former when the medium is suddenly switched to another sugar, galactose, suggesting that one bet is placed on cellobiose remaining the only available sugar, and another bet is placed on the return of a more preferred sugar [21]. A similar phenomenon is seen with *E*. *coli* populations shifted from glucose to gluconeogenic carbon sources [22]. The complex dynamics of sugar availability and response are only beginning to be explored. In *E*. *coli* it does appear that the population-level observation of a lag as cells shift from glucose to lactose obscures an underlying bimodality, wherein most cells lag but some cells do not [23]. However, responses to different sugars can be very different: whereas some sugars induce their utilization pathways in bimodal fashion, others show uniform induction or complex combinations of the two [24]. Moreover, complex environments might favor genetic diversification, as in the case of *E*. *coli* populations repeatedly allowed to consume medium containing glucose and acetate; two subpopulations that switch at different rates to acetate consumption when glucose is depleted are maintained by negative frequency-dependent selection [25].

More than 70 years after Monod introduced the diauxic shift, it remains a powerful example of how organisms cope with changing environments at the molecular, physiological, and evolutionary levels. Perhaps because the molecular underpinnings of the glucose to lactose shift in *E*. *coli* have become essential textbook knowledge, the prevailing view of the diauxic shift has remained one of tight regulation and uniform cell behavior. The time has come for this paradigm to shift. Recent work, including the three recent papers in *PLOS Biology* [7–9], has made clear that microbial populations live in diverse and changing environments that exert selective pressures that do not always favor perfect catabolite repression. Cells process signals about their environments to anticipate changes and, in the absence of reliable signals, populations hedge their bets. The imperative now is to uncover the molecular mechanisms of anticipation, memory, and bet hedging, as well as to venture out of the lab to study the ecological pressures that actually shape these mechanisms.
